# Pharmacotherapy for Irritable Bowel Syndrome

**DOI:** 10.3390/jcm6110101

**Published:** 2017-10-27

**Authors:** Michael Camilleri, Alexander C. Ford

**Affiliations:** 1 Clinical Enteric Neuroscience Translational and Epidemiological Research (C.E.N.T.E.R.) and Division of Gastroenterology and Hepatology, Mayo Clinic, 200 First Street S.W., Rochester, MN 55905, USA; 2 Leeds Institute of Biomedical and Clinical Sciences, University of Leeds and Leeds Gastroenterology Institute, Leeds Teaching Hospitals Trust, Leeds LS9 7TF, UK; alexf12399@yahoo.com

**Keywords:** diarrhea, constipation, pain, viscera, analgesia

## Abstract

Irritable bowel syndrome (IBS) is a disorder of the brain-gut axis; the pathophysiological mechanisms include altered colonic motility, bile acid metabolism, neurohormonal regulation, immune dysfunction, alterations in the epithelial barrier and secretory properties of the gut. This article reviews the mechanisms, efficacy, and safety of current pharmacotherapy, and medications that are in phase III trials for the treatment of IBS. There remains a significant unmet need for effective treatments—particularly for the pain component of IBS—although the introduction of drugs directed at secretion, motility and a non-absorbable antibiotic provide options for the bowel dysfunction in IBS.

## 1. Introduction

Numerous pathophysiological mechanisms are implicated in IBS and it is likely that individual or multiple disease processes in each individual patient lead to pain and diarrhoea, or pain and constipation. As a result, IBS treatment is often selected on an individual basis, and is targeted at the predominant or most troublesome symptom experienced by the patient, rather that attempting to modify the disease and the natural history of the disorder [1]. This highlights the need for future high-quality randomized controlled trials (RCTs) of interventions based on the pathophysiological mechanisms, and the opportunity to confirm the presence of those mechanisms based on validated, actionable biomarkers, such as abnormalities of colonic transit or bile acid metabolism [2,3]. Pharmacotherapy is one component of the treatment of IBS; other therapies can be considered either alone or in association with pharmacotherapy and these include lifestyle changes, dietary modifications, and psychological therapies, including relaxation and cognitive behavioral therapy.

An important component, and largely unmet need, in the treatment of IBS is pain. Sensory neurons reach the central nervous system via vagal, thoracolumbar and lumbosacral pathways. Parasympathetic afferents, comprising the majority of nerve fibers in the vagus and pelvic nerves, convey non-conscious sensory information, to the nucleus of the solitary tract in the brainstem. Visceral afferents course along sympathetic nerves, and convey painful stimuli to the spinal cord via the dorsal roots. These afferents are equipped with a variety of pro- and antinociceptive ion channels and receptors; the balance between pain sensing and suppressing signals finally determines the activation status of the nerve ending [4]. Important neurotransmitters involved in visceral sensation are 5-HT and neurokinins. Important channels mediating activation of afferent nerves are transient receptor potential (TRP) ion channels, and TRPV1, TRPM8, and TRPA1 all act as molecular detectors of thermal and chemical stimuli that activate sensory neurons to produce acute or persistent pain [5].

Conventionally, IBS is divided into subtypes according to the predominant stool pattern because this informs treatment options. Syntheses of the available literature have provided evidence-based treatment recommendations for the management of IBS based on these symptom subtypes [6,7,8,9] based on large high-quality trials, using Food and Drug Administration (FDA)-recommended endpoints to judge efficacy. However, for many of the more traditional therapies, the RCTs studying these agents were smaller, of lower quality, recruited heterogeneous groups of patients with IBS, and the endpoints used were of debatable validity [10].

For each drug class, we summarize mechanism, efficacy and safety of IBS (Table 1).

**Figure 1:**
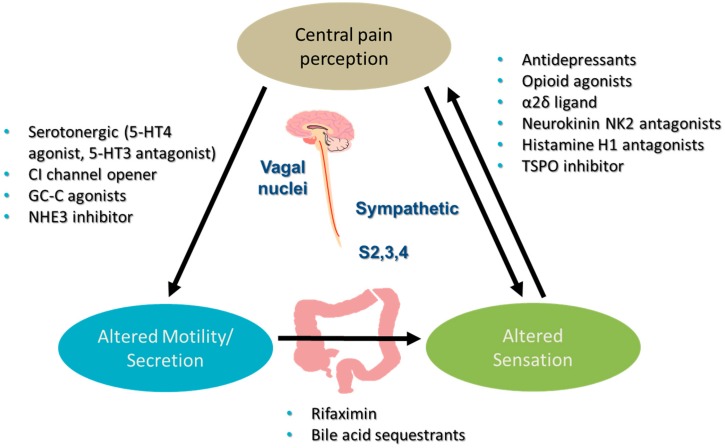
Pharmacotherapy in Irritable Bowel Syndrome. 5-HT = 5-hydroxy tryptamine; GC-C-guanylate cyclase C; NHE = sodium-hydrogen exchanger; S2, 3, 4 = sacral nerves 2, 3 and 4.

The predominant site of action of the different classes of drugs is shown in Figure 1.

## 2. Antispasmodic Drugs

### 2.1. Mechanism

Antispasmodics inhibit the action of acetylcholine at muscarinic receptors, or via blockade of calcium channels, on gastrointestinal (GI) smooth muscle. Otilonium bromide targets not only L- and T-type calcium channels, but also muscarinic type 2 and tachykinin NK2 receptors, possibly contributing to its increased efficacy. As a class, antispasmodics have been used in the treatment of IBS for many years, on the basis that a subgroup of patients with IBS have abnormal contractility of GI smooth muscle, and altered GI transit, and that this contributes to pain and disturbances in bowel habit (reviewed in [11]).

### 2.2. Efficacy

#### 2.2.1. Efficacy Focused on Systematic Reviews

A 2005 Cochrane review concluded that there was weak evidence for the benefit of some antispasmodics for abdominal pain and global symptom relief, although it was unclear which individual classes were effective [12].

A separate European systematic review in 2006 [13] identified nine placebo-controlled studies of antispasmodics in IBS with limitations identified due to lack of standardized diagnostic criteria, and low to intermediate quality since they were performed before the development of the Rome criteria for study design. Abdominal pain was significantly improved in 7 of 9 studies, bowel symptoms in 2 of 9, and 4 of 9 studies reported global symptom severity improvement.

A 2008 meta-analysis identified 22 separate RCTs involving 1778 patients, studying 12 different antispasmodic drugs [14]. Overall, as a class, antispasmodics were more effective than placebo, with a relative risk of remaining symptomatic of 0.68 (95% CI 0.57 to 0.71), and a number-needed-to-treat (NNT) of 5. Individual medications were efficacious when evaluated in subgroup analyses, with estimated NNTs as follows: hyoscine 3.5 (3 trials, 426 patients), otilonium 4.5 (4 trials, 435 patients), cimetropium 3 (3 trials, 158 patients), and pinaverium 3 (3 trials, 188 patients). However, significant heterogeneity, moderate methodological quality, and possible publication bias reduce confidence in the estimation of efficacy; in addition, there was no report of efficacy according to IBS subtype. Of the 12 agents studied, the strongest data were for otilonium bromide and pinaverium. It is also important to point out that most of the drugs studied in the trials included in this meta-analysis are licensed in other countries including many countries in Europe and Asia, and are not licensed for the treatment of IBS in the United States.

#### 2.2.2. Efficacy Focused on Otilonium Bromide Trials

Clavé et al. randomized 356 patients with all subtypes of IBS to either 40 mg otilonium bromide (OB) tid or placebo for 15 weeks [15]. Abdominal pain frequency score improvement by ≥1 pointwas higher with otilonium (69%) versus placebo (56%, *p* = 0.02). This was measured on a 4-level rating scale based on the number of pain episodes per week registered in the patient diary: 0 = 0 episode; 1 = 1–3 episodes, 2 = 4–7 episodes, 3 = 8 or more episodes. This effect was consistent across all IBS subtypes. Otilonium also reduced the frequency of episodes of abdominal pain, abdominal bloating, and improved global efficacy and probability of remaining relapse free during 10 weeks of follow up. However, there was no significant difference in quality of life.

The efficacy of OB in IBS has been confirmed in four studies [14], including significant improvement of abdominal pain and bloating severity with OB versus placebo [16] or reduction in the number of pain episodes and severity of abdominal distention, improved well-being and global assessment, but not in bowel symptoms [17]. A post-hoc analysis of symptom ratings found higher response rates with OB for a wide range of symptoms [18].

#### 2.2.3. Efficacy Focused on Pinaverium Trials

In an RCT of pinaverium conducted in 427 Chinese patients with IBS-D [19], 77.5% of patients receiving pinaverium had either a ≥30% reduction from baseline in abdominal pain or a ≥50% reduction in the number of days with at least one stool with a Bristol stool score ≥6 at week 4, compared with 33.5% with placebo (*p* < 0.001). The proportion of patients who achieved both endpoints at week 4, was also higher with pinaverium (38.1% vs. 16.7%, *p* < 0.001). This is the only RCT of antispasmodic drugs that utilizes an FDA-preferred endpoint for the treatment of IBS-D. However, these findings need to be replicated in other ethnic groups and in studies of a longer duration.

### 2.3. Safety

Side effects were significantly more frequent with antispasmodics compared with placebo, the commonest of which were dry mouth, dizziness, and blurred vision. Antispasmodics are generally well tolerated, apart from anticholinergics which can cause atropine-like side effects, including constipation [11].

## 3. Peppermint Oil

### 3.1. Mechanism

The major constituent of peppermint oil is menthol, which has antispasmodic properties. Menthol inhibits smooth muscle contractility in the GI tract by blocking calcium influx, via L-type calcium channels in the plasma membrane of smooth muscle cells [20,21]. Recent evidence has indicated that this menthol-induced analgesia is mediated by activation of the temperature sensing ion channel, TRPM8 [22]. This same receptor is expressed by nociceptive visceral afferents, where TRPM8 has anti-nociceptive properties. One can thus anticipate that peppermint oil, if delivered efficiently to these afferent nerve endings, may contribute to a better pain relief compared with standard antispasmodics.

### 3.2. Efficacy

In a meta-analysis from 2008 [14], peppermint oil was more effective than placebo in four trials, containing 392 patients with IBS, with a relative risk of remaining symptomatic of 0.43 (95% CI 0.32 to 0.59), and a NNT of 2.5. However, there was borderline heterogeneity between studies, and none of the trials were of high-quality, which may have led to an over-estimate of its efficacy. In addition, the effect of peppermint oil according to IBS subtype was not reported.

In a systematic review and meta-analysis of five randomized, controlled trials of an older formulation of peppermint oil that included 197 patients on the active treatment arm and 195 on placebo, the analysis favored peppermint oil (RR 2.23 (95% CI 1.78–2.81)) over placebo [23]. Peppermint oil was significantly superior to placebo for global improvement of IBS symptoms (5 studies) and improvement in abdominal pain (5 studies) [23]. Most of the clinical trials performed were however small in size and, therefore, lacked sufficient statistical power to draw definite conclusions.

A novel formulation, designed for sustained release in the small intestine, is now available for use in the US. In a 4-week trial of this formulation [24], comprising 72 patients with IBS-D or IBS-M, there was a 40% reduction in symptom scores from baseline with peppermint oil, compared with a 24% reduction with placebo, although there was no superiority over placebo for total IBS symptom score, but pain, bloating and urgency scores were reduced.

### 3.3. Safety

Peppermint oil can worsen gastroesophageal reflux symptoms and lead to heartburn, dry mouth, belching, a peppermint taste, and a peppermint smell [11].

## 4. Antidepressants

### 4.1. Mechanism

There is a convincing rationale for the potential of antidepressants in IBS. For example, co-existent psychological disorders are common among patients with IBS [25]; depression modifies the brain’s response to painful stimuli [26]; antidepressants have beneficial effects in chronic painful disorders [27,28]; and they affect GI motility, with tricyclic antidepressants (TCAs) prolonging orocecal and whole gut transit times, and selective serotonin re-uptake inhibitors (SSRIs) decreasing orocecal transit time [29]. It would therefore seem sensible to use TCAs in IBS-D, and SSRIs in IBS-C.

The mechanism of action of antidepressants in IBS remains uncertain, reduced activation of pain centers in the anterior cingulate cortex during painful rectal distension in patients with IBS by amitriptyline [30] suggests central effects on pain processing in addition to the effects on peripheral mechanisms that may influence sensation (such as colonic compliance and visceral afferent function).

### 4.2. Efficacy

An updated systematic review and meta-analysis [31] identified 17 separate trials of antidepressants with an overall beneficial effect on IBS symptoms: RR of remaining symptomatic 0.67 (95% CI 0.58 to 0.77), and an NNT of 4. However, only three of the RCTs were of high-quality—the majority of trials were conducted in secondary or tertiary care, and there was evidence of heterogeneity between studies and possible publication bias. In addition, two of the studies conducted in Iran, may have been atypical with placebo response rate of 14% [32], or “complete” response to amitriptyline of 63% [33], which seems unusually high. Therefore, the estimated NNT of 4 for the antidepressant class may be overestimated in this meta-analysis.

Antidepressant efficacy appears more convincing for TCAs, with an NNT of 4 and no heterogeneity between the 11 studies, compared with SSRIs with an NNT of 4 but significant heterogeneity between the seven trials. Seven RCTs (with 182 patients on antidepressants and 169 patients on placebo) reported the effect on abdominal pain, and the RR of abdominal pain persisting was significantly lower compared with placebo (0.62; 95% CI 0.43 to 0.88); however, there was considerable heterogeneity between studies (I^2^ = 72.4%). Effectiveness according to IBS subtype has only been assessed in two RCTs to date [32,33]. It is unclear whether the efficacy of antidepressants in IBS results from the treatment of co-existent depression. Three of the identified studies reported that there was no correlation between improvement in IBS symptoms and depression scores [34,35,36], and a fourth trial reported that the benefit of desipramine (a TCA) was greater in non-depressed individuals [37]. However, in an RCT by Ladabaum and colleagues [38], which excluded participants with depression, there was no benefit of citalopram. A 12-week, open-label trial of the SSRI, duloxetine, was conducted in 13 subjects with IBS and generalized anxiety disorder and showed improvement in overall and severity scales of IBS as well as symptoms of anxiety and QOL [39]. Effectiveness of antidepressants in relation to co-existent anxiety or other mental health conditions was seldom assessed in the literature. For example, beneficial therapeutic effect of citalopram was independent of effects on anxiety [35]. However, cognitive factors (sense of control over the condition, positive relationship with therapist or study coordinator, confidence in treatment, improvement in maladaptive cognitions, and quality of life during treatment) were all significant predictors of treatment response to medical and psychological treatments (deipramine, education and cognitive behavioral therapy) in functional bowel disorders, in contrast to demographic and other clinical variables which were not predictive [40].

### 4.3. Safety

Side effects were significantly more common with TCAs, with the most frequent being drowsiness and dry mouth. The meta-analysis by Ford et al. [30] documented that 31.3% of patients taking antidepressants complained of adverse effects compared with 16.5% of those given placebo (RR = 1.63, CI: 1.18–2.25). The number needed to harm was 9 (95% CI = 5–111).

Recent literature questions the safety of long-term use of antidepressants for non-psychiatric indications because of a possible link with dementia with some classes of psychotropic drugs [41,42], although causality has not been proven.

## 5. Drugs Acting on Opioid Receptors

### 5.1. Mechanism

Opioid receptor agonists slow GI and colonic transit, increase fluid absorption and reduce pain sensation—a review of the overall effects on different regions of the gut appears elsewhere [43].

### 5.2. Efficacy

Loperamide and diphenoxylate, μ-opioid agonists, are anti-diarrheal agents that have been used in IBS for many years [44]. However, this is based on limited evidence from rigorous RCTs. In one small trial of 21 patients with IBS-D, loperamide appeared to be beneficial, in terms of improved stool consistency, pain, and urgency [45]. In a second trial [46] conducted among 60 patients with either functional diarrhea or IBS (only 21 patients had both abdominal pain and disordered bowel habit), there was an improvement in stool frequency and consistency, as well as a reduction in the number of days with pain. A third trial [47] demonstrated benefit of loperamide in an unselected cohort of IBS patients with regard to stool frequency, stool consistency, and overall pain intensity, but with increased abdominal pain during the night. A position statement for the management of IBS suggested that there was insufficient evidence to recommend the use of loperamide [8], but the drug may be useful in clinical practice in those with debilitating diarrhea or urgency.

Eluxadoline is a novel κ-, and μ-opioid receptor agonist and δ-opioid receptor antagonist. Three large clinical trials, involving a total of over 3000 patients randomized to eluxadoline or placebo, have demonstrated efficacy of eluxadoline in the relief of diarrhea or the composite endpoint of diarrhea and pain over 12 weeks of treatment [48,49].

### 5.3. Safety

Adverse events with eluxadoline were chiefly nausea and headache, with rare cases of pancreatitis and sphincter of Oddi spasm [48,49]. The drug is now licensed for the treatment of IBS-D in the United States but the FDA recommends that patients with a history of biliary obstruction, cholecystectomy, pancreatitis, severe liver impairment, or severe constipation, and patients who consume more than three alcoholic drinks per day should not be prescribed eluxadoline (https://www.fda.gov/Drugs/DrugSafety/DrugSafetyPodcasts/ucm547907.htm).

## 6. 5-HT_3_ Receptor Antagonists

### 6.1. Mechanism

Serotonin, or 5-HT, is an important neurotransmitter in the brain and the enteric nervous system, with 90% of the body’s total store of 5-HT contained within the intestinal enterochromaffin cells [50,51]. Patients with IBS-D have increased postprandial plasma 5-HT, while those with IBS-C have reduced postprandial 5-HT levels [52]. Drugs that act on the 5-HT_3_ receptor, such as the antagonist alosetron [53], are known to retard colonic tranist. 5-HT_3_ receptors are also important mediators of visceral pain [54].

### 6.2. Efficacy

Several meta-analyses of RCTs have shown that the drug is effective [55,56] with an NNT of 8 for relief of abdominal pain and 4 for improvement in global symptoms. Alosetron is licensed for use in women with severe IBS-D in the United States, but is regulated by an FDA prescribing program.

Ramosetron has been used in the treatment of IBS-D in both men and women, with response rates respectively of 47% to 51%, compared with 27% to 32% with placebo (*p* < 0.001) [57,58], and it is now licensed for use in patients with IBS-D in Japan.

In a cross-over clinical trial [59] of 120 patients with IBS-D, ondansetron had a significant effect on stool consistency, as well as significant improvements in urgency, frequency of defecation, and bloating, but again no effect on pain. Constipation occurred in 9% of patients on ondansetron.

### 6.3. Safety

As a drug class, 5-HT3 antagonists can induce constipation, although this is usually manageable by titrating the dose; alosetron, but not other drugs in this class, is associated with ischemic colitis (~1:800 treated patients) [60].

## 7. Experimental Approaches Using Visceral Analgesics

### 7.1. Histamine H_1_ Receptor Antagonist, Ebastine

*Mechanism*: Mast cells and their mediators, in particular histamine, serotonin and proteases, are increasingly recognized as contributing to the pathogenesis of IBS [61]. Histamine is released by colonic biopsies from patients with IBS and induces visceral hypersensitivity to colorectal distention in murine models. Histamine sensitizes TRPV1 on neurons from murine dorsal root ganglia and on human submucosal neurons in rectal biopsies via activation of H_1_ receptors [62]. Moreover, supernatant from IBS biopsies sensitized murine DRG neurons, an effect also mediated via HRH1.

*Efficacy:* A clinical trial assessed 51 IBS patients who were treated with ebastine, a non-sedating antagonist of histamine H_1_ receptors. Ebastine reduced visceral hypersensitivity and overall IBS symptoms and abdominal pain in patients with IBS [62].

*Safety:* Headache, nausea, tiredness, and dry mouth are the most frequently reported adverse events on ebastine treatment but their incidence was similar to placebo-treated patients with seasonal and perennial rhinitis in 3 randomized, double-blind, multicenter clinical trials [63].

### 7.2. Neurokinin-_2_ Receptor Antagonist, Ibodutant

*Mechanism:* Neurokinins (NK, e.g., substance P) and NK_2_ receptors are abundantly expressed in the GI tract and mediate smooth muscle in the gut. NK_2_ receptor activation is also involved in stimulation of sensory nerves and activation of visceral reflexes.

*Efficacy:* In a phase 2, dose-finding study, the highly selective NK_2_ antagonist ibodutant, which has high oral bioavailability, improved pain severity in IBS-D, especially in those with a baseline score > 1 [64]. A more recent multi-national, phase 2, randomized, double-blind, placebo-controlled study in 559 patients showed a dose-dependent improvement in overall symptoms, abdominal pain and stool pattern in IBS-D in females, but not in males; the best efficacy was observed with a 10 mg dose [65].

*Safety*: The tolerability of the compound was reported to be excellent.

### 7.3. Selective Inhibitor of Translocator Protein TSPO

*Mechanism:* Translocator protein 18 kDa (TSPO) is a five-domain transmembrane protein that is highly expressed in steroid-producing tissues, including the glial cells within the brain. ONO-2952 is a novel and selective inhibitor of translocator protein 18 kDa that reduces stress-induced defecation and visceral hyperalgesia in rat models.

*Efficacy:* In a proof of concept, multicenter study of 200 patients who were on treatment for 4 weeks, there was significant improvement with the 60 mg dose (but not the 20 mg dose) in worst abdominal pain at week 3 [66]. There were also numerical, but not significant differences in abdominal pain, stool consistency or stool frequency during the other weeks of treatment.

*Safety:* There were no clinically significant adverse events related to the study drug.

## 8. GABAergic Agents

*Mechanism:* GABAergic agents are α2δ ligands that generally bind potently to an auxiliary protein associated with voltage-gated calcium channels, reducing depolarization-induced calcium influx at nerve terminals. This reduces the release of several excitatory neurotransmitters, including glutamate, noradrenaline, substance P, and calcitonin gene-related peptide (CGRP), which are involved in pain mechanisms.

*Efficacy:* Forty patients with IBS-D were randomized for a 5-day period to treatment with gabapentin, 300 mg/day and then 600 mg/day; rectal sensory thresholds were increased through attenuating rectal sensitivity to distension and enhancing rectal compliance [67]. Pregabalin has been tested in pharmacodynamic studies in healthy controls [68] and in patients with IBS, with significant pharmacodynamic effects on rectal or colonic compliance and sensation thresholds or ratings [69].

A preliminary report of a randomized, controlled clinical trial of pregabalin, 225 mg, in 85 patients with IBS reported lower average pain scores during weeks 9–12, and average symptom severity scores were lower with pregabalin than placebo [70].

*Safety:* There are insufficient data in the preliminary report [70] to assess safety in IBS patients. In the fibromyalgia literature, pregabalin, >300 mg dose, has been associated with somnolence, dizziness, and >7% weight gain in an analysis of 5 trials with 3808 patients [71].

## 9. Bile Acid Sequestrants

About 25% of patients with IBS-D have evidence of bile acid malabsorption based on ^75^SeHCAT scanning [72] or biochemical testing of serum or stool [73]. To date, there are no randomized, controlled trials of BA sequestrants in IBS. In a single center open-label trial of 10 days of 1875 mg twice daily colesevelam [74] in 12 patients with IBS-D and abnormal bile acid kinetics (increased fecal excretion of bile acids and fasting serum C4 suggesting a compensatory increase in the hepatic synthesis of bile acids), there was a reduction in stool consistency on the Bristol stool form scale. The number of bowel movements per week correlated inversely with the total bile acid sequestered into the stool, providing evidence for sequestration of bile acids being the mechanism for the observed improvement in diarrhea. In another open-label study, Bajor and colleagues [75] treated 27 patients with IBS-D and a ^75^SeHCAT retention <20% with colestipol 1 g BID. After 8 weeks of treatment, there were significant improvements in IBS symptom severity scores, stool frequency was reduced, and 15 (55.5%) of the 27 patients reported adequate relief of symptoms.

## 10. Antibiotics

### 10.1. Mechanism

Some patients with IBS may have underlying small intestinal bacterial overgrowth (SIBO), detected on hydrogen breath testing, and this can be reversed with non-absorbable antibiotics such as rifaximin [76]. However, the use of hydrogen breath testing as a basis to treat SIBO is controversial because of possible false positive results caused by rapid small bowel transit, unless there is concomitant measurement of the arrival into the colon of radiolabel added to the substrate, e.g., lactulose or glucose [77].

### 10.2. Efficacy

Rifaximin, a non-absorbable antibiotic, improved global symptoms and bloating in IBS in several trials, including 2 phase III, randomized, placebo-controlled trials [78,79] comprising more than 1200 patients with non-constipated IBS. Rifaximin, 550 mg three times daily for 2 weeks, led to significantly higher rates of adequate relief of global IBS symptoms and bloating with an NNT of 9–12.5; the effect on symptoms persisted out to 10 weeks post-treatment. However, stool consistency, number of bowel movements, and urgency were not improved.

A meta-analysis of five randomized controlled trials of rifaximin [80], comprising 1803 patients, reported similar efficacy with an NNT of 10 for improvement in global symptoms and bloating. A further trial has been conducted [81], in which 2579 patients received open-label rifaximin, 550 mg three times daily for 2 weeks. Among the 1074 patients who responded to treatment and were successfully followed, 59.2% had a recurrence of symptoms at a median of 10 weeks (range 6 to 24 weeks) post-treatment. They were then randomized to up to two repeat courses of rifaximin, 550 mg three times daily for 2 weeks each, separated by 10 weeks, or placebo, in a double-blind manner. Response rates were significantly higher with rifaximin after both the first and the second repeat treatments, and with a difference in the percentage of responders of only 8% relative to placebo. The FDA has approved the use of rifaximin for IBS-D patients with up to two repeat treatments in case of recurrence of symptoms. A small randomized, controlled study [82] appraised the potential mechanisms for the beneficial effect on symptoms in non-constipated IBS patients, and showed acceleration of ascending colon emptying and overall colonic transit at 48 h, which is paradoxical given the indication for this drug in the treatment of IBS-D. There were no differences from placebo in terms of effects on permeability, stool microbiome, or stool bile acids.

### 10.3. Safety

Adverse event rates were similar to those associated with placebo and there were no cases of *Clostridium difficile* [81]. Moreover, short-term, repeat treatment with rifaximin has no apparent long-term effect on stool microbial susceptibility to rifaximin, rifampin, and non-rifamycin antibiotics [83]. While long-term treatment with rifaximin is not the approved mode of administration and its safety in patients with IBS has not been demonstrated, it is reassuring to note that long-term use in patients with hepatic encephalopathy appears to be safe, and certainly safer than neomycin treatment [84].

## 11. Intestinal Secretagogues

### 11.1. Chloride Channel-Related

Lubiprostone, a prostaglandin derivative, acts on CIC-2 chloride channels on the apical membrane of the intestinal enterocyte. This leads to active chloride secretion with passive movement of sodium ions and water into the lumen—GI transit is accelerated and stools become looser. The drug has been studied at a dose of 8 mcg twice daily for 12 weeks in two large phase III trials including 1171 patients with IBS-C [85]. In a pooled analysis from both RCTs, response rates (at least moderate relief of global symptoms for 2 out of the 3 months of therapy) were 17.9% with lubiprostone, compared with 10.1% with placebo (*p* = 0.001). There were also improvements in abdominal pain scores, straining, and stool consistency, but no significant effect on quality of life. Nausea was the commonest side effect, experienced by 8% of patients.

Linaclotide is a minimally absorbed 14-amino acid peptide, which is a guanylate cyclase C receptor agonist. This increases intra-cellular cyclic guanosine monophosphate (cGMP), secretion of chloride and bicarbonate into the intestinal lumen, via the cystic fibrosis transmembrane regulator, and sodium and water secretion. The increase in cGMP may also have effects on sensory afferent neurons, leading to pain inhibition, an effect noted in the phase III clinical trials of the drug that were conducted in chronic idiopathic constipation [86], where abdominal discomfort and bloating improved significantly. In the two phase III trials of 290 mcg once daily conducted in IBS-C [87,88], response to therapy (≥30% decrease in pain, and an increase of ≥1 complete spontaneous bowel movement per week) was demonstrated in both trials and an NNT of 5 or 8 at 12 weeks. The main adverse event with linaclotide was diarrhea, occurring in almost 20% of participants in both studies.

Plecanatide is a 16-amino acid peptide analog of uroguanylin. Uroguanylin is an endogenous agonist that binds and activates guanylate cyclase-C (GC-C) receptors expressed in the epithelial lining of the GI mucosa in a pH-sensitive manner [89]. In addition to published efficacy in patients with chronic idiopathic constipation [90], there are preliminary reports of efficacy in patients with IBS-C [91]. Thus, two randomized trials involving 1135 patients (71.8% female) showed that plecanatide 3 mg and 6 mg were associated with significant differences compared with placebo in terms of overall responders (≥30% reduction in worst abdominal pain and an increase of ≥1 complete spontaneous bowel movement from baseline, in the same week, for ≥50% of the 12 treatment weeks). Responder rates in study one were: 3 mg, 30.2%, 6 mg, 29.5% compared with placebo 17.8%; and in study two: 3 mg, 21.5%, 6 mg, 24.0% compared with placebo, 14.2% (all comparisons with placebo *p* < 0.001).

Lubiprostone, linaclotide and plecanatide are all approved by the FDA for the treatment of chronic idiopathic constipation—in addition, lubiprostone and linaclotide are also approved for IBS-C, and plecanatide will be reviewed by the FDA for approval for the IBS-C indication in the first quarter of 2018.

### 11.2. Sodium-Hydrogen Exchanger

Tenapanor is a small-molecule inhibitor of the gastrointestinal sodium/hydrogen exchanger, NHE3, which results in increased intraluminal sodium and water excretion. A phase 2 dose-response study included 356 patients with IBS-C, of whom 305 completed the study [92]. Tenapanor, 50 mg b.i.d., compared to placebo was associated with increased responder rate for complete spontaneous bowel movement (CSBM) response (increase from baseline of ≥1 CSBM/week for ≥6/12 treatment weeks), composite response (CSBM and abdominal pain), and individual abdominal symptoms (pain, discomfort, bloating, cramping, and fullness). Diarrhea was the most frequent adverse event (50 mg b.i.d., 11.2%). The 5 mg and 20 mg b.i.d. doses were not significantly different from placebo.

## 12. 5-HT_4_ Receptor Agonists

As a class, 5-HT_4_ receptor agonists have demonstrated efficacy in patients with IBS-C. In a summary analysis of the major trials of tegaserod in patients with IBS in whom constipation was the predominant symptom, Layer et al. evaluated major clinical trials of tegaserod, which involved 8948 IBS patients [93]. Tegaserod was an effective treatment for IBS-C, providing statistically significant relief of overall and multiple individual IBS-C symptoms (abdominal pain/discomfort, bloating, and constipation) in both placebo-controlled and open-label settings. Repeat treatments with tegaserod were also shown to be effective, and tegaserod was associated with improvements in patients’ quality of life and work productivity [93]. Adverse effects associated with tegaserod were diarrhea, cramping, and cardiovascular AEs. Mosapride also accelerated gastric and small bowel transit time and improved symptoms in patients with IBS-C in a pilot, 10-patient study [94]. However, in a 12-month study of 69 patients, there were no significant improvements in overall IBS symptoms, specific symptoms (pain, bloating, stool frequency or consistency) or quality of life with mosapride over placebo [95].

Tegaserod and cisapride were “old generation” drugs in this class, with cardiovascular liabilities that have been resolved with newer medications in this class such as prucalopride, naronapride, velusetrag, and YKP10811. However, these medications have been tested predominantly in chronic idiopathic constipation where they have been shown to be efficacious based on systematic review [96] or a single center combined pharmacodynamic and patient response study [97].

## 13. Conclusions

At the present time, the treatment of IBS remains focused on treating the patient’s predominant, or most troublesome, symptom. However, the efficacy of most pharmacotherapies is modest, high-quality evidence for some is sparse, and none have been shown to alter the long-term natural history of the disorder. In the past, the treatment of IBS was an inexact science. However, therapies are being developed that target some of the important pathophysiological mechanisms, and the methods to plan and conduct high-quality, randomized, controlled trials are readily available. This augurs well for impactful treatments in the future.

## Figures and Tables

**Table 1:** Summary of current treatments and drugs in development for IBS.

Therapy	Mechanism of Action	Efficacy	Quality of Data	Adverse Events	Limitations of Data
Antispasmodic drugs	Smooth muscle relaxation	May be effective	Low	More likely with antispasmodics in a meta-analysis of 22 RCTs, particularly dry mouth, dizziness, and blurred vision	No high-quality trials, heterogeneity between studies, possible publication bias, and only a small number of RCTs assessing each individual antispasmodic
Peppermint oil	Smooth muscle relaxation	Effective	Moderate	No increase in adverse events in a meta-analysis of 4 RCTs	Heterogeneity between studies
Antidepressants	Central sensory modulation	Effective	Moderate	More likely with antidepressants in a meta-analysis of 17 RCTs, particularly dry mouth and drowsiness	Few high-quality trials, heterogeneity between studies, possible publication bias, and some atypical trials included
Ibodutant	Neurokinin NK_2_ antagonist	May be effective	Moderate	Promising visceral analgesic in a phase 2B trial	Awaiting phase 3 trials
Ebastine	Histamine H_1_ antagonist	May be effective	Low	Promising visceral analgesic in a single center trial	Awaiting phase 2B trials
TSPO inhibitor		May be effective	Low	Modest efficacy in a single proof of concept trial	Awaiting phase 2B trials
Loperamide	μ-opioid agonist	Unknown	Low	Limited data	Few RCTs, with a small number of participants, not all of whom had IBS
Eluxadoline	Mixed opioid receptor modulator	Effective	High.	Serious events included acute pancreatitis and sphincter of Oddi spasm. Nausea and headache commoner with active therapy	Only a modest benefit over placebo in published RCTs; no benefit over placebo in terms of abdominal pain
Alosetron, ramosetron, ondansetron	5-HT_3_ receptor antagonists	Effective	High	Serious events with alosetron included ischemic colitis and severe constipation. Ramosetron and ondansetron may be safer, although constipation commoner with active therapy.	Fewer RCTs of ramosetron and ondansetron; ondansetron may have no benefit over placebo in terms of abdominal pain
Cholestyramine, colestipol, colesevelam	Bile acid sequestrants	Unknown	Low	Limited data	No published RCTs
Rifaximin	Non-absorbable antibiotic	Effective	Moderate	No increase in adverse events in a meta-analysis of 5 RCTs	Only a modest benefit over placebo in published RCTs
Lubiprostone	Cl-C2 channel agonist	Effective	Moderate	Nausea commoner with active therapy, occurring in 8% of patients	Only a modest benefit over placebo in published RCTs
Linaclotide	GC-C receptor agonist	Effective	High	Diarrhea commoner with active therapy, occurring in 20% of pts	None
Plecanatide	GC-C receptor agonist	Effective	High	Diarrhea commoner with active therapy, occurring in ~6% of pts	None
Tenapanor	NHE_3_ inhibitor	Effective	Moderate	Diarrhea commoner with active therapy, occurring in 12% of pts	Awaiting phase 2B/3 trials
Prucalopride	5-HT_4_ receptor agonist	Effective	high	Diarrhea, cramping, and cardiovascular AEs with “old generation” drugs in this class	Data available for tegaserod and mosapride, not for “new generation” drugs in this class: prucalopride, naronapride, velusetrag, YKP10811

Cl-C2 = chloride channel 2; GC-C = guanylate cyclase C; 5-HT = 5-hydroxy tryptamine; NHE = sodium-hydrogen exchanger; RCT = randomized controlled trial; TSPO = translocator protein.
